# Tomato Fruit Development and Metabolism

**DOI:** 10.3389/fpls.2019.01554

**Published:** 2019-11-29

**Authors:** Muriel Quinet, Trinidad Angosto, Fernando J. Yuste-Lisbona, Rémi Blanchard-Gros, Servane Bigot, Juan-Pablo Martinez, Stanley Lutts

**Affiliations:** ^1^ Groupe de Recherche en Physiologie Végétale, Earth and Life Institute, Université Catholique de Louvain, Louvain-la-Neuve, Belgium; ^2^ Centro de Investigación en Biotecnología Agroalimentaria (BITAL), Universidad de Almería, Almería, Spain; ^3^ Instituto de Investigaciones Agropecuarias (INIA-La Cruz), La Cruz, Chile

**Keywords:** abiotic stress, fruit set, fruit ripening, genetic control, hormonal control, primary metabolism, secondary metabolism, *Solanum lycopersicum*

## Abstract

Tomato (*Solanum lycopersicum* L.) belongs to the Solanaceae family and is the second most important fruit or vegetable crop next to potato (*Solanum tuberosum* L.). It is cultivated for fresh fruit and processed products. Tomatoes contain many health-promoting compounds including vitamins, carotenoids, and phenolic compounds. In addition to its economic and nutritional importance, tomatoes have become the model for the study of fleshy fruit development. Tomato is a climacteric fruit and dramatic metabolic changes occur during its fruit development. In this review, we provide an overview of our current understanding of tomato fruit metabolism. We begin by detailing the genetic and hormonal control of fruit development and ripening, after which we document the primary metabolism of tomato fruits, with a special focus on sugar, organic acid, and amino acid metabolism. Links between primary and secondary metabolic pathways are further highlighted by the importance of pigments, flavonoids, and volatiles for tomato fruit quality. Finally, as tomato plants are sensitive to several abiotic stresses, we briefly summarize the effects of adverse environmental conditions on tomato fruit metabolism and quality.

## Introduction

Tomato (*Solanum lycopersicum* L.) is the second most important fruit or vegetable crop next to potato (*Solanum tuberosum* L.), with approximately 182.3 million tons of tomato fruits produced on 4.85 million ha each year (FAOSTAT, 2019). Asia accounts for 61.1% of global tomato production, while Europe, America, and Africa produced 13.5%, 13.4%, and 11.8% of the total tomato yield, respectively. Tomato yields are highly variable, ranging from more than 508 tons per ha in the Netherlands to fewer than 1.5 tons per ha in Somalia in 2017 (FAOSTAT, 2019), with an average global yield of 376 tons per ha. Tomato consumption is concentrated in China, India, North Africa, the Middle East, the US, and Brazil with tomato consumption per capita, ranging from 61.9 to 198.9 kg per capita (FAOSTAT, 2019). Tomato is a member of the Solanaceae family, which includes several other economically important crops such as potato, pepper (*Capsicum annuum* L.), and eggplant (*Solanum melongena* L.), representing one of the most valuable plant families for vegetable and fruit crops.

Tomatoes contain many health-promoting compounds and are easily integrated as a nutritious part of a balanced diet (Martí et al., 2016). In addition to consuming the fresh fruits, consumers use tomatoes in processed products such as soups, juices, and sauces (Krauss et al., 2006; Li et al., 2018b). Over the last decade, consumers have become more aware of foods as a source of health benefits and their roles in prevention of several chronic diseases and dysfunctions (Pem and Jeewon, 2015). Although a wealth of functional foodstuffs have been created to fulfil these requirements, it is important to note that the consumption of “conventional foods” such as fruits and vegetables is more effective for this purpose (Viuda-Martos et al., 2014).

The nutritional importance of tomatoes is largely explained by their various health-promoting compounds, including vitamins, carotenoids, and phenolic compounds (Raiola et al., 2014; Liu et al., 2016; Martí et al., 2016; Li et al., 2018b). These bioactive compounds have a wide range of physiological properties, including anti-inflammatory, anti-allergenic, antimicrobial, vasodilatory, antithrombotic, cardio-protective, and antioxidant effects (Raiola et al., 2014). Tomatoes are rich in carotenoids, representing the main source of lycopene in the human diet (Viuda-Martos et al., 2014). Carotenoids and polyphenolic compounds contribute to the nutritional value of tomatoes and improve their functional attributes and sensory qualities, including taste, aroma, and texture (Raiola et al., 2014; Tohge and Fernie, 2015; Martí et al., 2016). Tomatoes also have the naturally occurring antioxidants Vitamins C and E (Agarwal and Rao, 2000; Martí et al., 2016) as well as large amounts of metabolites, such as sucrose, hexoses, citrate, malate, and ascorbic acid (Li et al., 2018b).

Tomato fruit quality and metabolite biosynthesis are affected by plant growing conditions (Diouf et al., 2018). Tomato production is challenged by several problems around the world, including the scarcity of water resources, soil salinization, and other abiotic stresses (Fahad et al., 2017; Gharbi et al., 2017; Zhou et al., 2019). In particular, in countries with a Mediterranean climate, including some regions in southern Europe and North and South America, tomato cultivation is increasingly confronted with limiting conditions such as drought and salinity, which ultimately reduce the competitiveness of tomato farmers in these areas. This, in turn, impacts the integrity of the ecosystem, contributing to the relocation (abandonment) of rural sectors.

In addition to its economic and nutritional importance, tomatoes have become the model for the study of fleshy fruit development (Karlova et al., 2014; Kim et al., 2018; Li et al., 2018b). The entire tomato genome has been sequenced, serving as a rich genomic resource, and both genetic and physical maps and molecular markers are available for this species (The Tomato Genome Consortium, 2012; Suresh et al., 2014; Zhao et al., 2019). Moreover, a range of well-characterized monogenic mutants, TILLING populations, wild tomato species, recombinant inbred lines and genome editing tools are available (Eshed and Zamir, 1994; Minoia et al., 2010; Pérez-Martín et al., 2017; Li et al., 2018b; Martín-Pizarro and Posé, 2018; Tomato Genetics Resource Center, 2019; Rothan et al., 2019). Several databases contain gene expression analysis data (Fei et al., 2006; Suresh et al., 2014; Zouine et al., 2017; Shinozaki et al., 2018b), while recent progress in tomato metabolomics has provided substantial information about the primary and specialized metabolism of this species and the pathways involved in molecular biosynthesis and turnover (Luo, 2015; Tieman et al., 2017; Zhu et al., 2018).

Dramatic metabolic changes occur during tomato fruit development (Carrari and Fernie, 2006). Tomato is a climacteric fruit, meaning it undergoes a surge in respiration and ethylene production at the onset of ripening (Li et al., 2019a). As ripening progresses, tomato fruits transit from partially photosynthetic to true heterotrophic tissues through the parallel differentiation of chloroplasts into chromoplasts and the dominance of carotenoids and lycopene in the cells of the ripe fruits (Carrari and Fernie, 2006). The ripening process has evolved to make fruit palatable to the organisms that consume them and disperse their seeds. In doing so, ripening activates pathways that generally influence the levels of pigments, sugars, acids, and aroma-associated volatiles to make the fruit more appealing, while simultaneously promoting tissue softening and degradation to permit easier seed release (Matas et al., 2009).

In this review, we provide an overview of our current understanding of tomato fruit metabolism. We begin by detailing the genetic and hormonal control of fruit development and ripening, after which we document the primary metabolism of tomato fruits, with a special focus on sugar, organic acid, and amino acid metabolism. Links between primary and secondary metabolic pathways are further highlighted by the importance of pigments, flavonoids, and volatiles for tomato fruit quality. Finally, as tomato plants are sensitive to several abiotic stresses, we briefly summarize the effects of adverse environmental conditions on tomato fruit metabolism and quality.

## Genetic Regulation of the Development and Ripening of Tomato Fruit

### Fruit Set and Early Fruit Development

**Figure 1:**
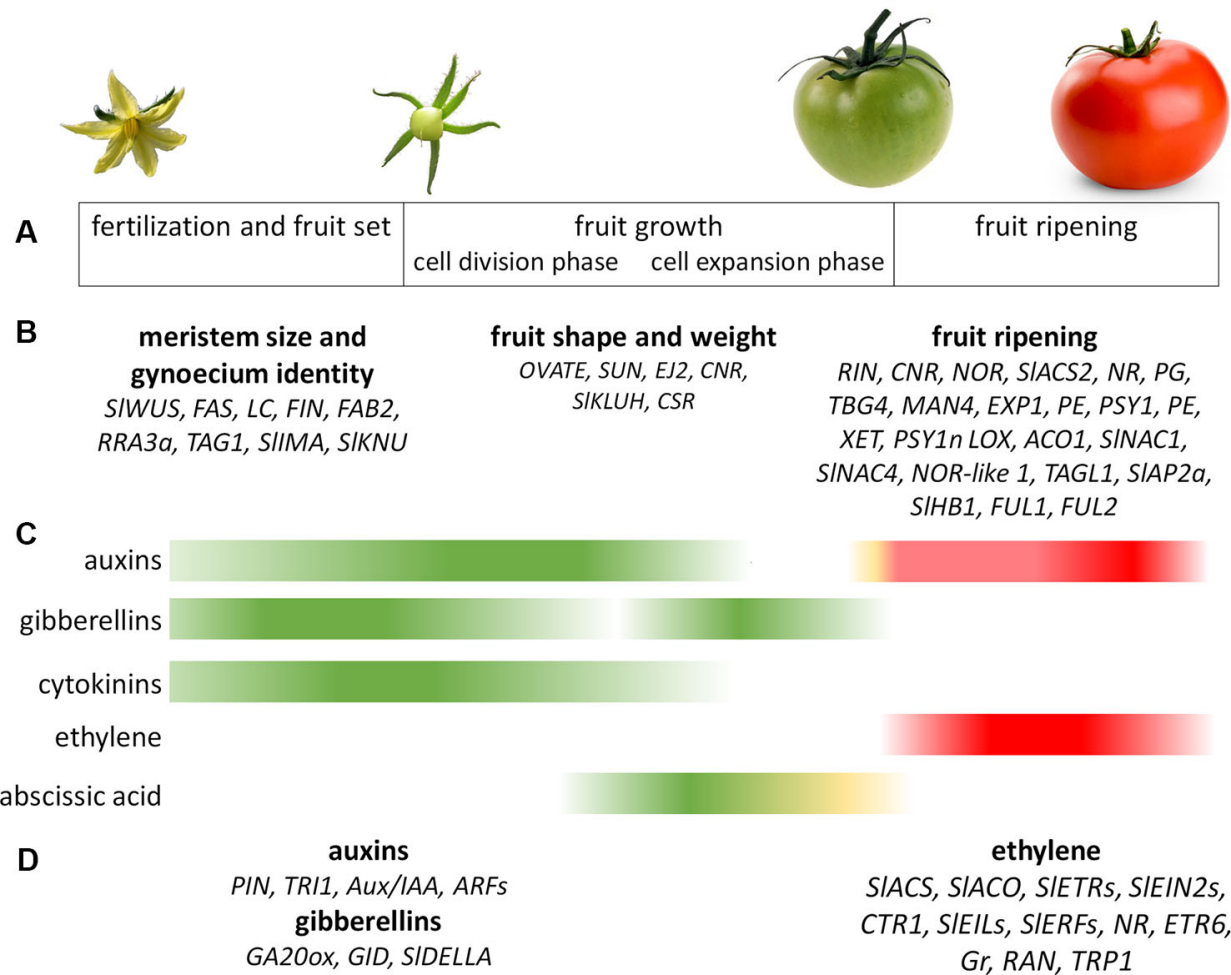
Genetic and hormonal control of tomato fruit development. **(A)** Main stages of tomato fruit development. **(B)** Genes involved in the control of tomato fruit development that are mentioned in this article. **(C)** Main hormones involved in tomato fruit development during fruit set and fruit growth (green) and fruit ripening (red). **(D)** Genes involved in the hormonal regulation of fruit development that are mentioned in this article. The Figure summarizes data collected by Gillaspy et al. (1993); Srivastava and Handa (2005); Karlova et al. (2014) and Obroucheva (2014).

The genetic regulation of fruit development begins in the floral meristem (FM), where the architecture and organization of this tissue is determined, and continues until the later developmental stages before fruit ripening (Gillaspy et al., 1993) (**Figures 1A**, **B**). At the initial stage of tomato fruit development, the CLAVATA-WUSCHEL (CLV-WUS) feedback loop controls meristem activity and regulates FM size, which in turn determines the final number of carpels in flowers and, hence, seed locules in fruits (Rodríguez-Leal et al., 2017). The signaling peptide CLV3 directly interacts with leucine-rich repeat receptor kinases, such as CLV1 or CLV2, to activate a signaling cascade that negatively regulates the stem cell-promoting transcription factor WUS (Somssich et al., 2016). Loss-of-function mutations in any of the *CLV* genes will therefore cause stem cell over proliferation, resulting in the development of extra floral organs and larger fruits (Xu et al., 2015; Rodríguez-Leal et al., 2017); for example, the joint action of the natural mutations *fasciated* (*fas*) and *locule number* (*lc*) gave rise to large-fruited cultivars, in contrast to the bilocular fruits of tomato wild species and most small-fruited varieties (Tanksley, 2004; Barrero et al., 2006). The *fas* mutation is a 294-kb inversion disrupting the tomato *CLV3* (*SlCLV3*) promoter (Xu et al., 2015), whereas *lc* is associated with two single-nucleotide polymorphisms in a putative CArG box regulatory element downstream of *WUS* (*SlWUS*) (Muños et al., 2011; van der Knaap et al., 2014). Furthermore, using forward genetics and CRISPR/Cas9 genome editing technology, Xu et al. (2015) identified the arabinosyltransferase genes *FASCIATED INFLORESCENCE* (*FIN*), *FASCIATED AND BRANCHED2* (*FAB2*), and *REDUCED RESIDUAL ARABINOSE 3a* (*RRA3a*) as new components of the CLV-WUS pathway. The SlCLV3 peptide must therefore be fully arabinosylated to maintain meristem size since the loss of an arabinosyltransferase cascade causes floral and fruit fasciation.

As the flower develops, the gynoecium is initiated in the fourth whorl to terminate FM activity. The MADS box transcription factor AGAMOUS (AG) is required to form the carpel primordium (Yanofsky et al., 1990). Consequently, the downregulation of *TOMATO AGAMOUS1* (*TAG1*), the tomato ortholog of *Arabidopsis thaliana AG*, gives rise to alterations in carpel development and determinacy by producing fruits that continue to develop in an indeterminate fashion (Pnueli et al., 1994; Pan et al., 2010; Gimenez et al., 2016). Furthermore, in *Arabidopsis*, *AG* turns off the stem cell maintenance program through the transcriptional repression of *WUS via* two different pathways: directly, by promoting the recruitment of Polycomb Group (PcG) proteins to methylate histone H3K27 at the *WUS* locus (Liu et al., 2011); and indirectly, by inducing the expression of a gene encoding the C2H2 zinc-finger protein KNUCKLES (KNU) (Sun et al., 2009). The induction of *KNU* expression by AG requires a time delay regulated by the epigenetic modification of histones at the *KNU* locus (Sun et al., 2014). Recently, Bollier et al. (2018) demonstrated that the AG-KNU-WUS pathway is conserved in Arabidopsis and tomato and regulates the timed termination of floral stem cell activity. In this context, the tomato mini zinc-finger protein INHIBITOR OF MERISTEM ACTIVITY (SlIMA) recruits SlKNU to form a transcriptional repressor complex together with TOPLESS and HISTONE DEACETYLASE19, which binds to the *SlWUS* locus to repress its transcription (Bollier et al., 2018). Additionally, it has been hypothesized that *lc* is a weak gain-of-function mutation that reduces or blocks the binding of TAG1 to the *SlWUS* 3′ regulatory region, which impairs the ability of TAG1 to repress *SlWUS*, resulting in the formation of larger fruits as a consequence of the development of extra carpels (van der Knaap et al., 2014).

The variation in tomato fruit morphology not only depends on CLV-WUS signaling pathway-related genes, but also on *OVATE* and *SUN*, which have a large effect on fruit shape (**Figure 1B**). The *ovate* null mutation gives rise to changes in cell division patterns during the earliest stages of gynoecium development, with more cells produced in the proximo-distal direction and fewer in the medio-lateral direction, causing the development of elongated fruits (Ku et al., 1999; Liu et al., 2002; Rodríguez et al., 2011). In contrast, the effect of *SUN* on fruit shape is most noticeable at flower anthesis, when it begins to increase cell division along the proximo-distal axis and cell elongation immediately after fertilization (Xiao et al., 2009; Wu et al., 2011; van der Knaap et al., 2014). Thus, a profound shift in the expression of genes involved in cell division, cell wall development, and patterning processes was observed in the elongating fruit tissues of the *sun* mutant (Clevenger et al., 2015). Moreover, the MADS box gene *ENHANCER OF J2* (*EJ2*) also seems to be involved in determining fruit shape; *ej2* knockout mutants develop slightly elongated fruits together with several pleiotropic effects, such as branched inflorescences and jointless pedicels (Soyk et al., 2017).

Among the fruit weight regulators, *CELL NUMBER REGULATOR* (*CNR*) was found to underlie the *fw2.2* quantitative trait locus (QTL), acting early during the development of the gynoecium to increase ovary size (Frary et al., 2000; Guo and Simmons, 2011) and enlarge the placenta and columella fruit tissues (Cong et al., 2002; Gonzalo et al., 2009). *SlKLUH* is the causal gene for the *fw3.2* QTL and encodes a CYP450 of the 78A class (Chakrabarti et al., 2013). One single-nucleotide polymorphism in the *SlKLUH* promoter leads to its enhanced expression in meristems and young flower bud tissues; however, the increased fruit weight of these mutant plants becomes evident only after fertilization. An increased number of cell layers in the pericarp gives rise to heavier fruits with a ripening delay, which has been hypothesized to be the result of the extension of the cell proliferation stage (Chakrabarti et al., 2013). Studies in *Arabidopsis* have suggested that KLUH is involved in generating a mobile growth-promoting signal, although its exact molecular and biochemical nature is yet to be deciphered (Anastasiou et al., 2007; Adamski et al., 2009). Cell expansion in the pericarp is responsible for the dramatic increase in fruit size from a 1- to 2-mm gynoecium to a 5- to 10-cm tomato fruit (Gillaspy et al., 1993; Xiao et al., 2009). The *CELL SIZE REGULATOR* (*CSR*) gene controls pericarp cell size and underlies the *fw11.3* QTL (Huang and van der Knaap, 2011; Mu et al., 2017). *CSR* expression is restricted to fruits, starting about 5 days after pollination and decreasing at the onset of ripening. Along with the increased cell size, coexpression studies suggest that *CSR* is also involved in shoot development and phloem/xylem histogenesis; however, the molecular function of *CSR* in controlling these developmental processes remains unclear (Mu et al., 2017).

### Fruit Ripening

At the end of fruit development, when seeds are mature and ready for dispersal, tomato fruits undergo ripening, a complex developmental program involving the coordinated regulation of numerous physiological and biochemical changes that determine flavor, color, texture, and aroma. These changes involve the up- or downregulation of numerous genes in various metabolic pathways (Alba et al., 2005; Fujisawa et al., 2011; Osorio et al., 2011). Multiple studies of the development and maturation of tomato fruits have facilitated the identification of specific genes that participate in ripening (Vrebalov et al., 2002; Manning et al., 2006; Giovannoni, 2007; Wang et al., 2009; Chung et al., 2010; Nashilevitz et al., 2010; Karlova et al., 2011; Pesaresi et al., 2014) (**Figure 1B**).

Tomatoes are classified as climacteric fruits, exhibiting a peak of respiration and ethylene production at the start of ripening (Alexander and Grierson, 2002). The biosynthesis and perception of ethylene are highly regulated, involving genes conserved in various plant taxa (Seymour et al., 2013). Some transcription factors modulate ethylene biosynthesis and signal transduction during fruit ripening, among which it is worth highlighting RIPENING INHIBITOR (RIN) (Vrebalov et al., 2002), COLORLESS NON-RIPENING (CNR) (Manning et al., 2006), and NON-RIPENING (NOR) (Yuan et al., 2016). RIN acts as the main regulator of fruit ripening, directly controlling the expression of target genes involved in a wide range of ripening-related events (Fujisawa et al., 2011; Qin et al., 2012). *RIN* encodes a SEPALLATA (SEP)-class MADS-box transcription factor (Vrebalov et al., 2002), which was previously considered to be an essential regulator of the induction of ripening (Vrebalov et al., 2002); however, its role in fruit ripening was recently reassessed following the publication of studies showing that *RIN*, although necessary to complete ripening, is not required for the initiation of this process (Ito et al., 2017). The *rin* mutant was found to be caused by the deletion of a genomic DNA fragment between *RIN* and *MACROCALYX* (*MC*), forming the chimeric gene *RIN-MC* (Vrebalov et al., 2002). *MC* affects inflorescence determinacy and sepal development (Vrebalov et al., 2002), and the *rin* mutant was found to be a gain-of-function mutant that produced a protein that actively represses ripening (Ito et al., 2008; Li et al., 2018a). RIN binds to the demethylated promoter regions of several genes, such as the ethylene biosynthesis genes *SlACS2* (*1-AMINOCYCLOPROPANE-1-CARBOXYLIC ACID SYNTHASE 2*), *SlACS4*, *SlACO1* (*ACC OXIDASE 1*), the ethylene receptor *NEVER RIPE* (*NR*), and others whose products are involved in fruit softening and the transcriptional regulation of cell wall hydrolases [*POLYGALACTURONASE* (*PG*), β*-GALACTOSIDASE4* (*TBG4*), *ENDO-*(*1,4*)*-*β*-* *MANNANASE4* (*MAN4*), and α*-EXPANSIN1* (*EXP1*)] (Klee and Tieman, 2002; Ito et al., 2008; Fujisawa et al., 2011; Martel et al., 2011; Shima et al., 2013; Ito et al., 2017).

RIN also positively stimulates the expression of *CNR* (Cardon et al., 1999; Manning et al., 2006). The *cnr* mutation is the result of a spontaneous epigenetic change that increases cytosine methylation in the promoter of a *SQUAMOSA* promoter-binding protein-encoding gene, which strongly decreases gene expression and produces colorless fruits with an altered pericarp texture (Manning et al., 2006). During ripening, the *CNR* promoter is progressively demethylated, but in *cnr* mutants, the promoter remains hypermethylated, preventing RIN from binding to it (Zhong et al., 2013). In addition, CNR was involved in the positive regulation of many ripening-related genes, including *PG*, *PECTINESTERASE* (*PE*), *XYLOGLUCAN ENDOTRANSGLYCOSYLASE* (*XET*), *PHYTOENE SYNTHASE1* (*PSY1*), *LIPOXYGENASE* (*LOX*), and *ACO1* (Eriksson et al., 2004).

The *nor* mutant exhibits abnormal ripening as a result of a 2-bp deletion in the *NOR* coding sequence, leading to the early termination of protein translation (Tigchelaar et al., 1973; Martel et al., 2011; Osorio et al., 2011). NOR encodes a NAC family transcription factor that regulates fruit ripening through a currently unclear mechanism, while mutations in this gene inhibit multiple metabolic processes and prolong fruit shelf life (Kumar et al., 2018). A study of the role of *NOR* and *RIN* in tomato fruit ripening confirmed that the *nor* mutation had a more global effect on ethylene/ripening-related gene expression than *rin*, suggesting that *NOR* might even act upstream of *RIN* in the transcriptional network controlling tomato fruit ripening (Osorio et al., 2011). In addition to *NOR*, three other NAC family genes, *SlNAC1*, *SlNAC4*, and *NOR-like1*, are known to be involved in the regulation of tomato fruit ripening (Ma et al., 2014; Zhu et al., 2014; Meng et al., 2016).

Other ripening factors, such as the MADS box TOMATO AGAMOUS-LIKE1 (TAGL1) (Vrebalov et al., 2002; Giménez et al., 2010), tomato APETALA2 (SlAP2a) (Karlova et al., 2011), and the tomato homeodomain leucine zipper homeobox protein SlHB1 (Lin et al., 2008), exercise their regulatory functions by interacting with RIN (Fujisawa et al., 2011; Qin et al., 2012; Seymour et al., 2013). *TAGL1* (also referred to as *ARLEQUIN* in some publications), a *PLENA* lineage gene orthologous to *Arabidopsis SHATTERPROOF1/2*, controls many aspects of tomato fruit ripening (Vrebalov et al., 2009; Garceau et al., 2017), including the direct activation of the expression of the ethylene biosynthesis gene *ACS2* (Itkin et al., 2009). Tomato fruits produced by *TAGL1-*silenced plants had defects in ripening without their floral organ specification being affected (Vrebalov et al., 2009; Giménez et al., 2010; Pan et al., 2010). Plants with reduced *TAGL1* expression produced fruits with a narrow pericarp and reduced firmness at the breaker stage, which remained yellow and produced significantly less ethylene than the control fruits (Vrebalov et al., 2009). The MADS box proteins TAGL1 and two homologs of FRUITFULL (FUL1/TDR4 and FUL2/MBP7) function as coregulators of RIN (Leseberg et al., 2008; Itkin et al., 2009; Vrebalov et al., 2009; Giménez et al., 2010; Martel et al., 2011; Bemer et al., 2012; Shima et al., 2013; Wang et al., 2014). Fujisawa et al. (2014) demonstrated that RIN, TAGL1, and the FUL homologs form a DNA-binding complex, probably a tetramer, which is believed to regulate tomato fruit ripening. The RIN and CNR regulators have been shown to function upstream of *SlAP2a* and to positively regulate its expression (Karlova et al., 2014), whereas *SlHB1* controls ethylene metabolism by binding to the regulatory regions of *ACO1* (Lin et al., 2008). On the other hand, transcriptomic studies have shown that *SlAP2a* participates in the control of fruit ripening as a negative regulator of several processes involved in ethylene biosynthesis, and signaling pathways, as well as in the differentiation of chromoplasts (Chung et al., 2010; Karlova et al., 2011).

## Hormonal Regulation of the Development and Ripening of Tomato Fruit

### Fruit Set and Early Fruit Development

Fruit set and fruit development are complex processes that require the coordination of different phytohormones (McAtee et al., 2013; Shinozaki et al., 2018b; Li et al., 2019b) (**Figures 1C, D**). From flower initiation to fertilization, the morphogenesis and growth of carpels and ovules require the spatial and temporal biosynthesis and action of auxins, cytokinins (CKs), and gibberellins (GAs) (Azzi et al., 2015). Shortly before anthesis, when the ovary has reached its mature size, abscisic acid (ABA) and ethylene work to stop growth within the ovary to maintain a temporally protected and dormant state (Gillaspy et al., 1993; Azzi et al., 2015). After the successful pollination and fertilization of the ovules, ovary growth resumes and the fruit and seeds develop concomitantly (Azzi et al., 2015). These changes are associated with a decrease in ABA and ethylene concentrations and an increase in auxin, GAs, and CKs (de Jong et al., 2009; McAtee et al., 2013; Shinozaki et al., 2015; Shinozaki et al., 2018a). GAs produced by pollen may increase auxin production in the ovary, which in turn may act as a signal for fruit set and the subsequent activation of cell division (Gillaspy et al., 1993; de Jong et al., 2009). Active fruit growth involving pericarp cell division and elongation is promoted by the biosynthesis of auxin in the developing seeds and GAs in the pericarp (Obroucheva, 2014). Auxins and GAs appear to be the predominant hormones required for tomato fruit initiation in response to fertilization, since the exogenous application of both hormones leads to fruit initiation and parthenocarpic development (de Jong et al., 2009). CKs, ethylene, ABA, brassinosteroids, and polyamines (PAs) have also been shown to play a role in fruit formation, but this is currently less well documented (Srivastava and Handa, 2005; McAtee et al., 2013; Azzi et al., 2015; Shinozaki et al., 2015; Liu et al., 2018; Shinozaki et al., 2018a).

In tomato, early fruit development is governed by the allocation of auxin to tissues and cells, which initiates signal transduction pathways (Azzi et al., 2015). The PIN-FORMED (PIN) auxin efflux transport proteins were shown to be involved in fruit set and early tomato fruit development (Mounet et al., 2012; Pattison and Catalá, 2012). Silencing *SlPIN4* resulted in the production of small parthenocarpic fruits exhibiting precocious development (Mounet et al., 2012). The auxin signaling pathway involves an auxin receptor called TRANSPORT INHIBITOR RESPONSE1 (TIR1) (Azzi et al., 2015). In the presence of auxin, TIR1 recruits the transcriptional repressors *AUXIN/INDOLE-3-ACETIC ACID* (*Aux/IAA*) and triggers their degradation by the 26S proteasome (Azzi et al., 2015), releasing the Aux/IAA-bound auxin response factors (ARFs) and initiating the auxin response through auxin-responsive element-mediated gene transcription (Azzi et al., 2015). In tomato, the misexpression of *TIR1* and specific members of the *Aux/IAA* and *ARF* gene family alters the normal flower-to-fruit transition and results in parthenocarpic fruit production (de Jong et al., 2009; Ren et al., 2011; Mounet et al., 2012; Azzi et al., 2015). However, *Aux/IAA* and *ARF* genes may have opposing functions to *TIR* regarding fruit set; the transcript abundance of *SlIAA9* and *SlARF7* decreased in *SlTIR1-*overexpressing plants, which resulted in the formation of seedless fruit (Ren and Wang, 2016; Goldental-Cohen et al., 2017). The silencing of the Aux/IAA transcriptional repressor *SlIAA17* resulted in larger fruits with thicker pericarp tissues, a phenotype caused by enhanced cell expansion (Su et al., 2014). Ren and Wang (2016) showed that *SlTIR* was regulated by GAs, auxins, ABA, and ethylene, suggesting that TIR may be a key mediator of the crosstalk between auxin and other phytohormones. The SlARF7/SllAA9 complex also mediates crosstalk between auxin and GA pathways to regulate fruit initiation through their interaction with the GA-signaling repressor SlDELLA (Hu et al., 2018). SlARF7/SllAA9 complex and SlDELLA antagonistically regulate genes involved in auxin and GA metabolism while they additively coregulate genes involved in fruit growth (Hu et al., 2018).

Indeed, auxins do not act alone to trigger fruit development and fruit set; these processes are partly mediated by GAs, as part of a complex hormonal cross-talk with auxin (de Jong et al., 2009; McAtee et al., 2013; Azzi et al., 2015). Pollination triggers the upregulation of transcripts encoding GA 20-oxidases (GA20ox), which biosynthesize active GA1 and GA4 (Azzi et al., 2015). It was suggested that the expression of more than one *GA20ox* gene is required to control fruit set in tomato because the silencing of individual *GA20ox* genes did not strongly affect fruit set or development (Xiao et al., 2006; Olimpieri et al., 2011; Azzi et al., 2015). Despite this, the heterologous overexpression of citrus *CgGA20ox1* in tomato resulted in an elevated GA4 content and parthenocarpic fruit development, demonstrating the influence of GA and GA20ox activity on fruit set and development (García-Hurtado et al., 2012). The GA signal transduction pathway requires the recognition of GA by its receptor, GA INSENSITIVE DWARF1 (GID1) (Azzi et al., 2015). The GID1-GA complex interacts with the nuclear repressor DELLA to target it for ubiquitin-dependent proteolytic degradation by the 26S proteasome (Azzi et al., 2015). This removes the repression of the GA-responsive genes, which are then able to initiate GA signal transduction. Consistent with this, the silencing of the *SlDELLA* gene in tomato resulted in small, facultative parthenocarpic fruits with an elongated shape (Martí et al., 2007). The *procera* (*pro*) mutant, which carries a point mutation in the GRAS region of *SlDELLA*, has also very strong parthenocarpic capacity and shows enhanced growth of preanthesis ovaries (Jones, 1987; Carrera et al., 2012; Shinozaki et al., 2018c). The parthenocarpic capacity of *pro* is mainly associated with changes in the expression of genes involved in GA and auxin pathways (Carrera et al., 2012). A new *SlDELLA* mutant containing a single nucleotide substitution, *procera2* (*pro2*), has been recently identified and shows a potential for high fruit yield in both optimal and unfavorable growing conditions due to its facultative parthenocarpic capacity (Shinozaki et al., 2018c). Parthenocarpy is indeed an attractive trait for fruit production (Shinozaki et al., 2018c).

As mentioned previously, other phytohormones are involved in fruit set and growth. A number of ABA-deficient mutants have provided valuable insights into the role of ABA in fruit growth (Azzi et al., 2015). Phenotypic characterization of the ABA biosynthesis *not/flc* double mutant showed that its small fruits had considerably reduced ABA levels and smaller cell sizes, especially within the pericarp (Nitsch et al., 2012). It was suggested that ABA stimulates fruit growth by restricting the level of ethylene in normal fruits (Azzi et al., 2015), which may indeed induce fruit set as tomato plants treated with the ethylene action inhibitor 1-methylcyclopropene (1-MCP) produce parthenocarpic fruits (Shinozaki et al., 2015). In the same way, tomato plants carrying either of two allelic mutations in *ETHYLENE RECEPTOR1* (*Sletr1-1* or *Sletr1-2*) were insensitive to ethylene, resulting in parthenocarpy (Shinozaki et al., 2015; Shinozaki et al., 2018a). Ethylene is involved in the senescence of unpollinated ovaries and prevents fruit set by downregulating GA accumulation, acting downstream of auxin and upstream of GA in the control of fruit set (Shinozaki et al., 2018a). Exogenous CK application induces parthenocarpic fruits (Matsuo et al., 2012; Ding et al., 2013), suggesting a role for CKs during tomato fruit initiation. Cytokinins induce parthenocarpy in tomato partially through modulation of GA and auxin metabolisms (Ding et al., 2013). Moreover, transcriptomic and metabolomic studies showed that although CKs mainly control cell division during tomato fruit development, they also play a critical role in fruit-set and early growth of tomato fruits (Mariotti et al., 2011; Matsuo et al., 2012). A key role for PAs during fruit set was also suggested, with tomato genes encoding enzymes involved in PA biosynthesis, such as arginine/ornithine decarboxylase (ADC/ODC) and spermine synthase (SPMS), suggested to be particularly important during the process of fruit setting (Liu et al., 2018).

### Fruit Ripening

Fruit ripening has been widely studied in tomato, with ethylene known to play a key role in this process (Osorio et al., 2013; Seymour et al., 2013; Liu et al., 2015; Borghesi et al., 2016; Shinozaki et al., 2018b; Li et al., 2019a) (**Figures 1C, D**). Two systems of ethylene biosynthesis have been proposed in climacteric fruits (McMurchie et al., 1972): System 1 is responsible for producing basal ethylene levels during fruit growth and is ethylene autoinhibitory, while system 2 operates during climacteric ripening and is autocatalytic (Liu et al., 2015). At the onset of ripening, an increase in ethylene is observed in mature green tomatoes, resulting in an eventual 100- to 300-fold increase in the ethylene concentration during fruit ripening (Karlova et al., 2014; Li et al., 2019a). Ethylene initiates a cascade of changes, which culminate in the transformation of the hard, unpalatable green tomato into an attractive, brightly colored succulent and nutritious fruit (Giovannoni, 2004; Li et al., 2019a).

Ethylene signaling can be regulated at several levels, including ethylene biosynthesis and its perception (Karlova et al., 2014; Mata et al., 2018; Li et al., 2019a). Ethylene biosynthesis involves multiple aminocyclopropane-1-carboxylic acid (ACC) synthase and ACC oxidase enzymes and genes (Osorio et al., 2013; Karlova et al., 2014; Kou et al., 2016; Li et al., 2019a). Fourteen putative *ACS* genes and six *ACO* genes have been identified in the tomato genome (Liu et al., 2015). Among them, it has been proposed that *SlACS2*, *SlACS4*, *SlACO1*, *SlACO2*, and *SlACO4* play important roles in ethylene production during tomato fruit maturation (Cara and Giovannoni, 2008; Liu et al., 2015). Some transcription factors are known to act upstream of the ethylene biosynthesis genes to regulate fruit ripening, including RIN, SlHB-1, and the NAC transcription factors SNAC4 and SNAC9 (Liu et al., 2015; Kou et al., 2016).

Ethylene perception is mediated through ethylene receptors encoded by *ETHYLENE RESPONSE* (*ETR*) genes, which activate a signal transduction cascade through the release of the block on *ETHYLENE INSENSITIVE2* (*EIN2*) exerted by *CONSTITUTIVE TRIPLE RESPONSE1* (*CTR1*) (Karlova et al., 2014; Liu et al., 2015; Mata et al., 2018; Li et al., 2019a). Seven *ETR* genes and four *CTR1* homologs have been identified in tomato thus far, all of which control ethylene sensitivity by balancing the turnover of the components of the ethylene signaling pathway, combining positive and negative feedback (Liu et al., 2015; Mata et al., 2018). This release then activates the *EIN3/EIN3-like* (*EIL*) primary transcription factor genes, resulting in the expression of secondary transcription factor genes encoding the ethylene response factors (ERFs) (Karlova et al., 2014; Liu et al., 2015; Mata et al., 2018). The final result of this signaling pathway is the transcriptional regulation of the target genes by the EILs or ERFs (Karlova et al., 2014). Some of the *ERF* genes have been characterized in tomato, including *SlERF1*, *SlERF.B3*, and *SlERF6* (Li et al., 2007; Liu et al., 2013; Karlova et al., 2014), but many of their functions and ethylene-responsive target genes remain unknown (Li et al., 2019a). Six *EIL* genes have been identified in tomato, although *SlEIL5* and *SlEIL6* may not be involved in tomato ripening (Liu et al., 2015). Several genes that regulate tomato ripening through the transduction of ethylene signals have been identified (Karlova et al., 2014), including the ethylene receptor genes *NR*, *ETR6*, and *GREEN-RIPE* (*Gr*) (Yen et al., 1995; Barry and Giovannoni, 2006; Kevany et al., 2007). Two other proteins, RESPONSE TO ANTAGONIST1 (RAN1) and TETRATRICOPEPTIDE REPEAT1 (TRP1), also play important roles at the receptor levels (Liu et al., 2015).

Ripening is also influenced by the balance of other hormones, including ABA, auxin, and the brassinosteroids (Seymour et al., 2013; Karlova et al., 2014; Liu et al., 2015; Shinozaki et al., 2018b; Li et al., 2019a; Shin et al., 2019). ABA is known to promote ripening, whereas auxin seems to have an antagonistic effect (Liu et al., 2015). ABA is a key intermediate regulator of tomato fruit ripening, and its levels change according to fruit development stages (Zhang et al., 2009; Borghesi et al., 2016). In tomato, the suppression of the gene that catalyzes the first step in ABA biosynthesis [9-cis-epoxy carotenoid dioxygenase (NCED1)] results in the downregulation of some ripening-related cell wall genes, such as those encoding polygalacturonase and pectin methylesterase, promoting an increase in firmness and a longer shelf life (Sun et al., 2012). ABA interacts with ethylene signaling; the expression of genes involved in ethylene biosynthesis are induced by exogenous ABA (Liu et al., 2015).

Low levels of auxins are also required at the onset of ripening, and auxin signaling declines at this stage (Gillaspy et al., 1993; Karlova et al., 2014; Shin et al., 2019); however, it seems that the ratio between indole acetic acid (IAA) and its conjugated forms is more important than the level of free IAA for the regulation of tomato ripening (Karlova et al., 2014). Indeed, the decrease of free IAA at the onset of ripening is associated with an increase in its conjugated form, IAA-Asp (Buta and Spaulding, 1994; Karlova et al., 2014). SlSAUR69 is involved in the decrease of auxin levels and/or signaling in the pericarp tissue at the onset of fruit ripening *via* the repression of polar auxin transport (Shin et al., 2019). *ARF* genes are also involved in fruit ripening; the downregulation of *SlARF4* or *SlARF2* resulted in fruits with dramatic ripening defects (Jones et al., 2002; Karlova et al., 2014; Hao et al., 2015). Auxin–ethylene interactions are crucial for the fruit ripening process, although the molecular basis of the regulatory network is still relatively unclear (Li et al., 2017; Shin et al., 2019). An antagonistic effect between auxin and ethylene has been observed during the ripening of tomatoes (Li et al., 2017), with ethylene inhibiting auxin transport, metabolism, and signaling processes, while auxin represses the expression of genes involved in ethylene biosynthesis and signaling (Chaabouni et al., 2009; Liu et al., 2015; Li et al., 2016a; Li et al., 2017). Moreover, both auxin and ethylene differentially regulate CK metabolism and signaling processes during tomato ripening (Li et al., 2017).

Brassinosteroids might also be involved in tomato ripening, as exogenous applications of this hormone can promote ripening and ethylene production in tomatoes (Karlova et al., 2014). PAs are also actively involved in climacteric fruit ripening (Liu et al., 2018); for example, putrescine levels progressively increase during fruit maturation and peak in ripe tomatoes, while spermine and spermidine levels decrease gradually until the fruits are fully ripe (Tsaniklidis et al., 2016; Liu et al., 2018). Moreover, although the expression levels of *SPMS*, *ADC*, and *ODC* were minimal during the fruit ripening process, the *SPDS* genes may play an important role during tomato fruit ripening (Liu et al., 2018).

Phytohormones also play a key role in the regulation of tomato fruit metabolism and quality (Van Meulebroek et al., 2015; Cruz et al., 2018; Li et al., 2019b). The hormones discussed above all contribute to the metabolism of tomato fruits, although ABA and ethylene play the most important roles (Li et al., 2019b). ABA had a greater effect on the regulation of the primary metabolism, while ethylene plays an important role in the transition of primary to secondary metabolism in tomatoes (Li et al., 2019b). Regarding secondary metabolism, ethylene and auxins were described as the most important regulators of carotenoid biosynthesis during tomato fruit ripening (Van Meulebroek et al., 2015; Cruz et al., 2018).

## Primary Metabolism in Tomato Fruit

**Figure 2:**
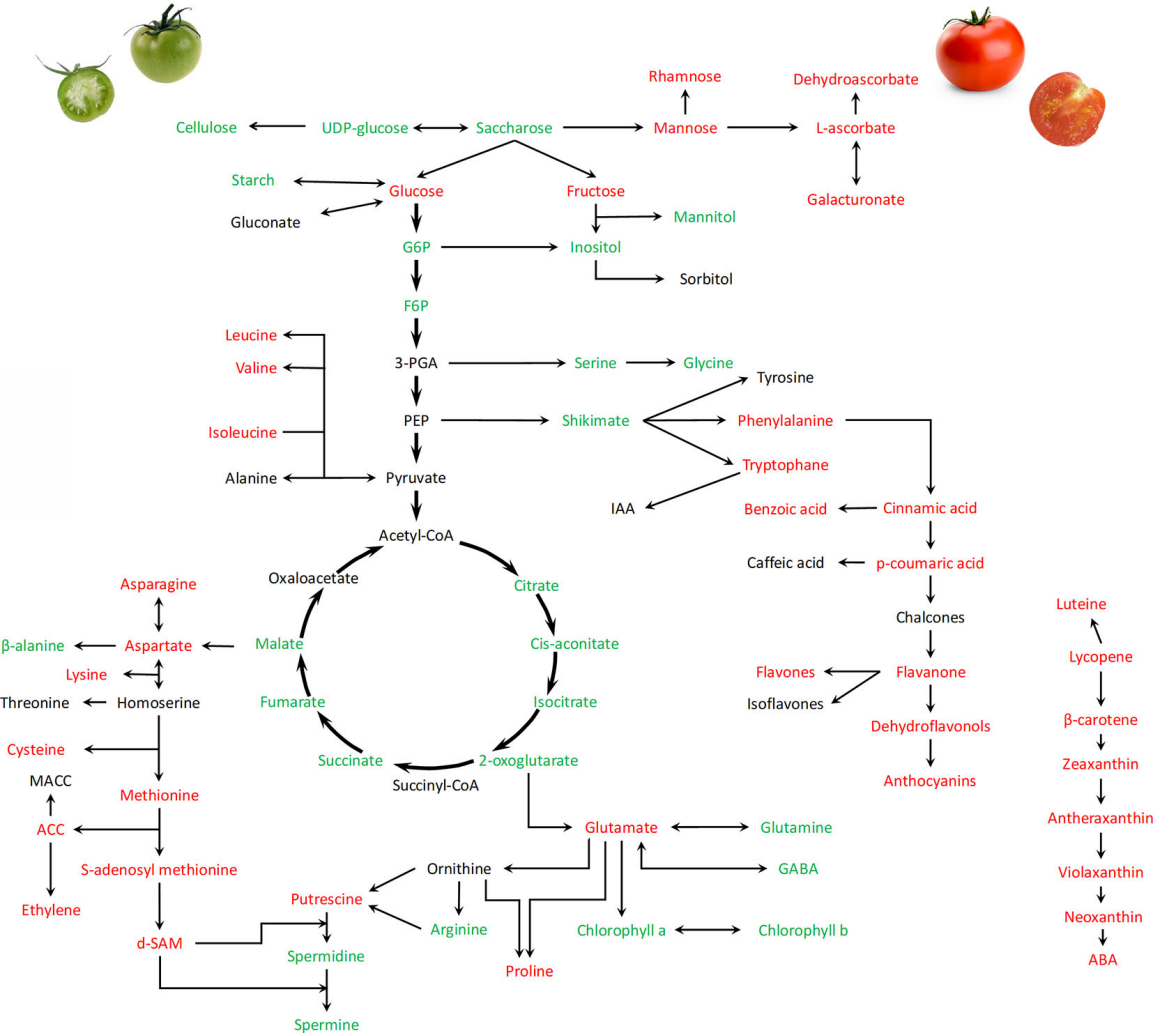
Global overview of metabolic changes occurring during the transition from green expanding fruit to ripening processes (from 30 DAA to 60 DAA) in tomato fruit. Names of metabolites in red, green and black indicate increase, decrease or no changes, respectively. Metabolites are analyzed mainly in pericarps. The Figure summarizes data collected by Carrari and Fernie (2006); Gilbert (2009); Mounet et al. (2009); Centeno et al. (2011); Beauvoit et al. (2014); Biais et al. (2014), Van Meulebroek et al. (2015), Van de Poel et al. (2012), and Zhao et al. (2018).

Development of the tomato fleshy fruit occurs in three distinct phases : i) cell division phase occurs in the early days following fertilization until 10 DAA ii) cell expansion (from 10 DAA to 40 DAA) and iii) fruit ripening and maturation (**Figure 1A**). During this evolution, tomato fruits follows a transition from partially photosynthetic to complete heterotrophic metabolism. Typical morphophysiological steps are considered and include immature, mature green, breaker, pink and red ripe fruits. Although the fruit ripening is an important step determining the fruit quality and nutritional values, recent works provided evidences that the early fruit development also assumes key roles for acquisition of quality traits, including the accumulation of sugars and organic acids (Carrari and Fernie, 2006; Beauvoit et al., 2014; Biais et al., 2014; Bauchet et al., 2017). Postgenomic approaches including analyses of fruit transcriptomes, proteomes, and metabolomes as well as multilevel studies integrating enzyme profiling generated a large set of useful data improving our knowledge on the regulation of metabolites turnover during tomato fruit development (Mounet et al., 2009; Centeno et al., 2011; Van de Poel et al., 2012; Van Meulebroek et al., 2015). Hierarchical clustering performed by Biais et al. (2014) revealed tight associations between enzyme activities and developmental phase and concluded that metabolites are more sensitive to growth conditions than enzyme activities. A global overview of the main recorded changes in metabolites recorded during fruit transition from green to red mature fruits is provided in **Figure 2**.

### Carbohydrate Metabolism

#### Immature Green Fruit Photosynthesis

Sugars are closely related to fruit yield and quality. In tomato fruits, sugars provide sweetness and are important for the generation of turgor pressure to promote cell expansion (Kanayama, 2017). Sugars also act as signal molecules controlling fruit development and metabolism. Green fruits remain able to perform photosynthesis which can produce up to 20% of the fruit photosynthetates, the remaining part being imported by source leaves (Pesaresi et al., 2014). The light harvesting electron transfer and CO_2_ fixation proteins are conserved in their active state in green fruit tissues (Matas et al., 2011). Fruit chloroplasts contain sufficient amounts of plastocyanin, ferredoxins, Rieske proteins, cytochrome *f* and cytochrome *b_559_* and ribulose-1,5-biphosphate carboxylase activity is detected in the fruits (Hetherington et al., 1998). The triose phosphate and glucose phosphate transporters are active in the tomato chloroplasts. Unexpectedly, genes associated with photosynthesis are highly expressed in the locule which is in fact the main site of respiration (Lemaire-Chamley et al., 2005).

Nevertheless, the importance of green fruit photosynthesis is still a matter of debate. According to Carrara et al. (2001), tomato fruits do not show signs of CO_2_ fixation, even if photochemical activity is detectable and an effective electron transport observed. Xu et al. (1997) reported that a small fruit (fresh weight lower than 10 g) is able to perform a gross photosynthesis equivalent to a 3-cm^2^ leaf blade but that this activity rapidly decreases thereafter: in heavier fruits, gross photosynthesis decreases to negligible values. These authors even assume that the aim of the photosynthetic process in maturing fruit is mainly to delete CO_2_ produced by respiration rather than contributing to photosynthate production. Kahlau and Bock (2008) showed that RNA, translation and protein accumulation downregulation was observed for all plastid-encoded photosynthesis genes already in the green fruit. Hetherington et al. (1998) however demonstrated that all truss tissues, including fruits, are quite active photosynthetically. These authors interestingly demonstrated that the relative contribution of the fruit versus the leaf photosynthesis for fruit photosynthate accumulation tend to narrow under low light intensities.

A fruit specific antisense inhibition of the chloroplastic fructose 1,6-biphosphatase (FBPase) led to an obvious decrease in final weight of ripe fruits (Obiadalla-Ali et al., 2004) while, conversely, tomato lines with a fruit specific reduction in the expression of glutamate-1-semialdehyde aminotransferase (GSA) and thus a lower level of chlorophyll and photosynthetic rate, remained unaffected in terms of fruit weight (Lytovchenko et al., 2011). Ntagkas et al. (2019) recently demonstrated that phosynthetically active fruits able to respond to light may trigger ascorbate synthesis while non-photosynthetic red maturing fruits are unable to produce this antioxidant in response to light.

Auxin plays an important role for determining final fruit stage through the control of cell division and cell expansion. Auxin-responsive factors (ARF) can either activate or repress transcription of auxin-responsive genes. Combined metabolomics and transcriptomic studies of plants deficient in the expression of the tomato *Aux/IAA* transcription factor *IAA9* suggest a role for photosynthesis in the initiation of fruit development (Wang et al., 2009). Downregulation of *SlARF4* enhanced fruit firmness and increased chlorophyll content in green fruits in relation to an increased number of chloroplasts (Guillon et al., 2008). SlARF4 also has a direct impact on fruit sugar metabolism: the *SlARF4* underexpression tomato lines accumulated more starch at early stages of fruit development associated with an improved photochemical efficiency (Sagar et al., 2013). Moreover, *SlARF4* is highly expressed in the pericarp tissues of immature fruits and undergoes decline at the onset of ripening. Down-regulated tomatoes also present a higher starch content than the wild type in developing fruits which is directly related to up-regulation of several genes and enzyme activities involved in starch biosynthesis (Sagar et al., 2013).

Plastid numbers and chlorophyll content in fruits are positively correlated with photosynthesis and photosynthate accumulation and both are influenced by numerous environmental and genetic factors. In tomato fruits, the *GOLDEN2-LIKE* (*GLK*) transcription factor induces the expression of numerous genes related to chloroplast differentiation and photosynthesis (Powell et al., 2012). The genome of *S. lycopersicum* possesses two copies of this gene: *SlGLK1* is predominantly expressed in the leaves while *SlGLK2* is expressed in the fruits, especially in the area of pedicel junction (Nguyen et al., 2014). A latitudinal gradient of *SlGLK2* expression induces a typical uneven coloration in ripe fruit *SlGLK2* is preferentially expressed in the shoulder of the fruit (Sagar et al., 2013). Sl-GLK2 belongs to the GARP subfamily of the myb transcription factor and is encoded by the *UNIFORM (U)* gene (Powell et al., 2012). The *u* mutation has been widely selected in modern tomato varieties which consequently exhibit a uniform ripening attractive to consumers and suitable for industrial processing. This mutant contains less sugar and chloroplasts present a lower number of thylakoid grana. According to Nadakuduti et al. (2014), some class I *KNOTTED1-LIKE HOMEOBOX* gene (*TKN2* and *TKN4*) also influence chloroplast development in tomato fruits and act upstream of SlGLK2. A dominant gain-of-function mutation of *TKN2* induces ectopic fruit chloroplast development that resembles *SlGLK2* overexpression. More recently, Lupi et al. (2019) demonstrated that *SlGLK2* expression is partly regulated by a phytochrome-mediated light perception. Auxin appears as a negative regulator of *SlGLK2* expression and SlGLK2 enhances cytokinin responsiveness. This study also demonstrated that SlGLK2 enhances tocopherol and total soluble solid through amylase stimulation, so that selection of the *u* mutation in commercial varieties probably inadvertently compromise ripe fruit quality.

#### Sugar Unloading in Fruits

Sugar unloading in tomato fruit is a controlled process and its pattern is not constant during the fruit development. In green developing fruits, sugar is mainly unloaded *via* the symplasm. Numerous plasmodesmata and cell connections are present at this stage (Ruan and Patrick, 1995) but then are progressively lost. During this early phase of development, only a small amount of sucrose is unloaded by the apoplastic invertase and transported into the fruit cells by hexose transporters (Nguyen-Quoc and Foyer, 2001; Beckles et al., 2012). Although it has been demonstrated that sucrose unloads in tomato pericarp until 35 DAA, a precocious role for apoplastic invertase has however been postulated on the basis of kinetics properties explaining a moderate QTL for Brix index (Fridman et al., 2004).

#### Sugar Metabolism At the Cell Division Stage

In growing fruits, sucrose represents less than 1% DW while fructose and glucose are the main accumulated soluble sugars (25 and 22% DW; Gilbert, 2009). Glucose and fructose content strongly increased during early fruit developmental phase. Most studies until recent year have focused on the ripe stage but omics analysis need to be conducted throughout fruit development since several interactions may occur between the different stages (Kanayama, 2017). In green fruits, hexose phosphates are mainly used for starch synthesis until 13 DPA. Starch accumulation in pericarp and columella tissues at this early stage is a key factor determining the final soluble solid content of mature fruits (Carrari and Fernie, 2006).

The sink strength of a developing fruit depends on both sink activity and sink size, the latter being a function of both the number and the size of the fruit cells. According to Kataoka et al. (2009), gibberellic acid just after anthesis can promote an increased sink size of individual pericarp cell through the activation of vacuolar acid invertase and neutral invertase. During the cell division phase, a high rate of mitotic activity is observed and the final cell number is determined at the end of this period. It is, at least partly, influenced by endoreduplication processes, seed number and hormonal cues. During cell division, enzymes involved in glycolysis (especially gluokinase and fructokinase) are activated. According to Biais et al. (2014), Glc-6-P is accumulating during this phase, and maintenance of a low ATP to ADP ratio and high hexose-P results in high flux through glycolysis. Pyruvate kinase and tricarboxylic acid cycle enzymes also exhibit high activities, indicating that ATP production as a priority. Beauvoit et al. (2014) postulated that the close match of the catalytic capacity to flux needs may be partly due to protein neosynthesis occuring during the early cell division phase, although protein-protein interactions and post-translational modifications may modulate enzyme *V*_max_ even if enzyme content remains constant.

At the end of the cell division phase, most soluble sugars accumulated in the vacuoles, together with malic and citric acid (Carrari and Fernie, 2006; Centeno et al., 2011). The osmotic potential of the vacuole consequently dropped to about ‑0.6 MPa and triggers water inflow in the dividing cells. Cytosolic sucrose synthase (SuSy) is involved in sucrose cleavage at the cell division stage. According to Nguyen-Quoc and Foyer (2001) cell vacuoles at this stage accumulate high concentration of hexose (up to 100 µmol g^‑1^ FW) and contain equal amounts of glucose and fructose. This implies that soluble sugars must be transported to the vacuoles by specific transporters. Vacuolar proton ATPase (V-ATPase) and vacuolar proton pyrophosphatase (V-PPase) generate the electrochemical gradient to transport sugar to the vacuolar compartment (Kanayama, 2017). Amemiya et al. (2006) showed that fruit specific V-ATPase suppression in antisense-transgenic tomato reduces fruit growth and seed formation. It is noteworthy that the highest expression of the *V-PPase* gene was observed during the cell division stage and not during latter stages of fruit development (Kanayama, 2017). Sucrose loading into the vacuole by the sucrose antiport–transporter is an efficient component of vacuolar storage and there is no requirement for sucrose hydrolysis to allow vacuolar loading or unloading. The fact that regulation of sugar transporters may be influenced by endogenous sugar levels through kinases provide an additional level of complexity regarding carbohydrate subcellular distribution at the end of the cell dividing phase (Lecourieux et al., 2010).

Beside Susy, acid invertase (AI; EC 3.2.1.26) may also be involved in sucrose cleavage and this implies that sucrolytic activity occurs within the vacuole and not only in the cytosol. According to Beauvoit et al. (2014), AI even assumes most of the sucrose cleavage in dividing cells while cytosolic neutral invertase (NI) and SuSy are mainly involved during the following cell expansion phase.

Although sucrose-phosphate-synthase (SPS : 2.4.1.14) activity remains low throughout the fruit development, it may significantly contribute to sucrose re-synthesis in the cytosol, inducing a « futile » cycle between sucrose and hexose characterized by a continuous sugar exchange between cytosol and vacuoles (sucrose influx and hexose efflux) (Nguyen-Quoc and Foyer, 2001). The extent of such resynthesis however remains limited from a quantitative point of view and never exceeds 10% of the cleavage (Beauvoit et al., 2014). Seeds may somewhat control the expression of genes coding for UGPase and SPS : Rounis et al. (2015) found drastic differences in transcript accumulation and enzyme activities of both UGPase and SPS between seeded and parthenocarpic fruits but only minor differences were recorded for sugar levels.

#### Sugar Metabolism During the Cell Expansion Phase

Mounet et al. (2009) explored transcriptional and metabolic changes in expanding fruit tissues (12–35 DAA) using multivariate analysis and gene-metabolites correlation networks. These authors demonstrated that cell expansion during fruit development proceeds differently in mesocarp and locular tissues which clearly differ in their metabolic composition. Mesocarp represent approximatively 50% (w/v) of the fruit fresh weight and its quantitative importance remains stable throughout fruit development while the locular tissues strongly develop reaching 23% (w/v) of the fruit fresh weight at the mature green stage. Some soluble sugars (mainly Suc and UDP-Glc) are most abundant in locular tissues at the end of the cell expansion phase while others such as hexoses mainly accumulate in the mesocarp. Beside differences in terms of distribution, discrepancies may also result from the mode of expression of enzyme activities or metabolites concentration. Biais et al. (2014) indeed estimated that expressing enzyme activities per protein content minimizes the influence of vacuolar expansion comparatively to an expression on a fresh weight basis.

The cell expansion phase itself is commonly divided in two distinct steps corresponding to « early » and « late » expansion. During the early cell expansion, enzymes involved in the middle part of the glycolysis (NAD-GAPDH, P6K, enolase [EC 4.2.1.11)] are activated in a coordinated way. The main enzyme controlling starch synthesis (ADP-Glc pyrophoshorylase) is also activated in order to produce ADP-Glc for starch synthesis. Sucrose synthase activity presented its highest value during this phase and could be involved in providing UDP-Glc for cell wall cellulose synthesis. Cell expansion is mainly driven by the hexose content. Hexose accumulation in the vacuole is responsible for at least 50% of the fruit osmotic potential during the time course of cell expansion. The crucial role of hexose in cell expansion may thus explain the small fruit size produced by shaded plants.

Some enzymes exhibit a high activity during the late elongation phase and culminate at the green mature stage. This is the case for phosphoglucoisomerase (PGI; EC 5.3.1.9), ATP-phosphofructokinase (PFK; EC 2.7.1.11) and for UDP-Glc pyrophosphorylase (UGPase; EC 2.7.7.9). These enzymes are involved in recycling of hexose-P issued from starch degradation (Carrari et al., 2007). Starch accumulation in the fruit occurs during the early expansion phase while net starch degradation occurs during the late cell expansion phase. Nevertheless, all enzymes required for starch synthesis and degradation are present in the fruits at all developmental stages and there is a continuous starch synthesis and breakdown in tomato fruits. The most important enzyme for starch degradation in fruit is starch phosphorylase which produces G1P while amylase activity remains rather low (Yelle et al., 1991). Beside regulation of ADP-Glc pyrophosphorylase, the concentration of hexose phosphate in the amyloplasts and the rate of hexose phosphate exchange between cytosol and amyloplast constitute major control points to regulate the balance between starch synthesis and starch degradation (Nguyen-Quoc and Foyer, 2001; Centeno et al., 2011).

SlARF4 represses the expression of SlAGPase gene (Sagar et al., 2013). Other transcription factors play key roles in the regulation of gene expression during cell expansion phase. Mounet et al. (2009) reported important roles for zinc finger proteins, MYB, bZIP, an ERF and a NAC transcription factors. The homeobox-Leu zipper protein HAT22 appears to be implicated in the complex regulation of the metabolic shift occurring between fruit early development and subsequent ripening. Some transcription factors assume important roles in the mesocarp while others are more specifically acting in the locular tissues (Lemaire-Chamley et al., 2005; Mintz-Oron et al., 2008; Mounet et al., 2009). Sugar signaling during the cell expansion phase may involve direct sugar-binding: hexokinase is acting as sugar sensor with dual independent functions in hexose phosphorylation and glucose sensing. Sugar signaling may also involve upstream open reading frame as reported for the sucrose-induced repression of translation in which the translation of the normal ORF of a bZIP transcription factor is repressed by sucrose (Kanayama, 2017). Sagor et al. (2016) expressed a tomato homolog of the bZIP gene lacking the uORF in fruit using a ripe fruit specific E8 promoter and strongly increased the fruit sugar concentration in the transgenic lines.

Both cell division and cell expansion phases imply the regulation of the cell wall metabolism, which also directly influences the fruit firmness and texture. Cell wall polysaccharides largely derive from sugar and sugar phosphates, and in tomato fleshy fruits mainly formed by unlignified parenchyma cells, pectic and hemicellulose polysaccharides account for nearly 95% of the cell wall. Regulation of the cell wall-related enzymes are however mainly studied in relation to the ripening phase of tomato fruit development.

#### Sugar Metabolism During Repining Phase and Putative Interest of Wild-Related Tomato Species

Ripening phase involves both catabolism and accumulation of key metabolites. During ripening, fruit weight still slightly increases and hexoses exhibit their highest concentration. Total protein content also increases and enzymes involved in TCA cycle and glycolysis strongly increased while glucokinase and fructokinase activities decreased. Degradation of starch hence becomes the main source of hexose-P used as substrate for respiration (Carrari and Fernie, 2006; Beckles et al., 2012; Biais et al., 2014). Sucrose-phosphate-synthase activity, which remains low during the previous expanding phases, significantly increased at the beginning of ripening phase (Biais et al., 2014). Accumulation of sucrose, however, remains limited since invertase activities also increased during ripening in the cultivated tomato species *S. lycopersicum* (Yelle et al., 1991). According to Bastías et al. (2011), ABA which increases before ethylene at the early beginning of maturation phase may be involved in stimulating the expression of genes coding acid vacuolar invertase. The ABA-responsive element binding factor SlAREB1 is indeed present in the fruit pericarp at the end of the mature green stage (Yáñez et al., 2009) and plays an important role for up-regulation of genes involved in sugar metabolism during ripening. During the breaker stage, chlorophyll content strongly declines and the dedifferentiation of chloroplasts in chromoplasts occur under the control of anterograd and retrograd mechanisms leading to the breakdown of starch granules and lysis of thylakoid membrane (Pesaresi et al., 2014). Cell walls are then degraded as a consequence of activation of rhamnogalacturonase and β-galactosidase which depolymerize branched pectins resistant to attack by endo-polygalacturonase (Carrari and Fernie, 2006). Pectin methylesterase catalyses de-esterification of pectin and are encoded by three genes, one being fruit specific and involved in shelf-life of tomato upon storage at room temperature. Fruit softening is also determined by cellulase (endo-β-1,4 glucanases) and by xyloglucan endotransglucosylase (Jiang et al., 2019).

Fructose is sweeter than other sugars and metabolic engineering was therefore specifically performed using fructokinase targets to increase fructose content in commercial tomato fruits (Odanaka et al., 2002; Kanayama, 2017). According to Schaffer et al. (1998), the trait of high fructose to glucose is independently inherited from that of sucrose accumulation. Numerous wild species differ from domesticated tomato cultivars and contain high TSS (Total Soluble Solid, a convenient proxy for sugar content) (more than 10% against 4–6% for *S. lycopersicum*). These wild species often present an increased import of sugar from source leaves, especially during the latter stage of development. Some of them (*Solanum chmielewskii, Solanum peruvianum, Solanum neorickiim*, and *Solanum habrochaites*) store large amounts of sucrose and present constitutively low invertase activities (Miron and Schaffer, 1991). Others (*Solanum cheesmanii, Solanum pennellii*, and *Solanum pimpinelifolium*) accumulate mainly glucose and fructose in relation to a high apoplastic invertase in the columella which increases the sugar gradient with the phloem (Beckles et al., 2012). Introgression lines thus constitute convenient tools to investigate the control of sugar content (Eshed and Zamir, 1994; Gur and Zamir, 2004). The line IL8-3 contains a single short segment from *S. pennellii* in the *S. lycopersicum* background. This promising line contains a high level of sugar resulting from an increased hexose content, probably as a consequence of a high activity of ADP-glucose pyrophosphorylase leading to accumulation of starch during the middle part of development, followed by an active starch remobilization during ripening (Ikeda et al., 2016).

Beside structural enzymes involved in sugar metabolism in fruits, sugar transporters also appear to play a key role in soluble sugar profile (Schroeder et al., 2013). This is especially the case for members of the *SWEET* gene family: the expression pattern of those genes frequently coincides with sugar accumulation pattern in tomato fruit (Feng et al., 2015). Two interacting chromosomal regions introgressed from the inedible *S. habrochaites* present an almost 3-fold epistatic increase in the fructose to glucose ratio in mature fruits (Levin et al., 2000). More recently, Shammai et al. (2018) reported that introgressions of the *Fgr^H^* allele from *S. habrochaite*s into cultivated tomato increased the fructose to glucose ratio of the ripe fruit. These authors clearly demonstrated that the *SlFgr* gene encodes a plasma membrane-localized glucose efflux transporter of the *SWEET* family. Its overexpression in transgenic tomato plants strongly reduced glucose concentration and increased fructose:glucose ratio. Interestingly, no clear impact of the *Fgr* gene overexpression on the expression of sugar metabolizing genes was recorded and the relationship between glucose efflux and fructose increase still remains an open question.

### Organic Acid Metabolism

Organic acid content in fruits is one of the most important properties from a commercial point of view and have a strong influence on the sensorial qualities of the product. Acid taste in tomato is attributed to citric and malic acid which constitute together more than 90% of the total pool of organic acid in harvestable fruits (Bastías et al., 2011). High sugar content and relatively high acid content are required for a favorable taste. High level of acids with low level of sugar will produce a tart tomato, while high levels of sugars and low acids will result in a bland taste (Davies and Hobson, 1981).

The cell division phase is characterized by very high rates of organic acids accumulation (from 2 to 5 nmol min^‑1^ g^‑1^ FW) between 4 and 15 DAA according to Beauvoit et al. (2014). It is consequently tempting to speculate that such a high level of accumulation contribute with soluble sugars to decrease the cell water potential allowing water uptake. However, beside this osmotic function, organic acids are also of paramount importance at the cellular level for various biochemical pathways. According to Carrari and Fernie (2006), manipulation of central organic acids is a promising approach to improve tomato fruit yield.

During the cell expansion phase, clear differences were recorded between locular and mesocarp tissues since most organic acids were more abundant in the former than in the latter, and this is especially the case for citrate and malate (Mounet et al., 2009). According to this study, among genes related to organic acid metabolism, 13 were differentially expressed in the two types of tissues. In both tissues, however, organic acid concentration increased between 20 and 35 DAA, mainly in locular tissues and this was correlated with an increased expression of gene coding for aconitase, a key enzyme involved in TCA cycle.

At the ripening stage, tomato climacteric fruits strongly increase ethylene synthesis and respiration, although both subsequently decreased during post-climacteric storage (Van de Poel et al., 2012). Increasing respiration implies hastening of the TCA cycle. Before the ethylene burst, a transient increase in ABA may induce an accumulation of citric and malic enzymes. At the beginning of the ripening phase, fruit preferentially accumulates citrate through stimulation of citrate synthase and the expression of a gene encoding mitochondrial citrate synthase is upregulated by SlAREB1 (Bastías et al., 2011).

Centeno et al. (2011) experimentally decreased the activities of mitochondrial malate dehydrogenase or fumarase *via* targeted fruit-specific antisense approach in tomato. These authors demonstrated that the line containing higher concentration of malate exhibited a lower starch accumulation during the cell expansion phase and lower soluble sugars at harvest. Although modification of organic acid content in the mitochondria could be relevant from modification in the TCA cycle, it has to be mentioned that mitochondrial pool represents only a small portion of the total cellular organic acid. According to Centeno et al. (2011), correlation between malate and starch concentration could be related to an altered redox status of the AGPase protein allowing an allosteric enhancement of its maximal catalytic activity.

During the ripening stage, phosphoenolpyruvate carboxykinase (PEPCK; which was almost undetectable in green fruits) is suspected to act in the dissimilation of malate/citrate to provide sugar through neoglucogenesis. This hypothesis was confirmed by Huang et al. (2015) who analyzed the effect of an excessive PEPCK in transgenic lines overexpression SlPEPCK by either the constitutive CaMV35S or the fruit-specific *E8* promoter. Soluble sugars increased while malate content decreased in both lines, confirming the participation of gluconeogenesis in sugar/acid metabolism during fruit ripening. Similarly, Schouten et al. (2016) recently confirmed that an important part of malate is converted to hexose

### Amino Acids Metabolism

Total concentration of free amino acids in tomato fruits varies between 2.0 and 2.5% on a dry weight basis. The most quantitatively important are Glu, Asp and GABA (γ-aminobutiric acid) (Sorrequieta et al., 2010; Snowden et al., 2015). GABA is a four carbon non-protein amino acid which assumes important functional properties in reducing blood pressure in the human body (Zhao et al., 2018). It is also an important metabolite in plants and control cytosolic pH under acid load *via* the GABA shunt pathway. It is present at high concentration at the green mature stage but then progressively declines during ripening processes (Klee and Giovannoni, 2011). Threonine also declines during ripening and could be metabolized to pyruvate involved with glyceraldehyde 3-phosphate in the synthesis off isopentenyl pyrophosphate acting as a precursor of carotenoids. Most of the other free amino acids increased during ripening while the protein content decreased in relation to an increment in exopeptidase activity and non-specific protease activity pattern (Sorrequieta et al., 2010).

The recorded increase is especially important for glutamate whose concentration may be as high as 10 mmol kg^‑1^ FW in mature fruits. Such an increase is partly due to stimulation of glutamate dehydrogenase (aminating reaction) and α-ketoglutarate-dependent γ-aminobutyrate transaminase. Cultivated *S. lycopersicum* has quite higher glutamate content than wild species (Schauer et al., 2005). Since glutamate is a direct precursor of chlorophyll, its accumulation in ripening fruit may be, at least partly, regarded as the consequence of downregulation of chlorophyll synthesis. Mature green fruit contain Fd-GOGAT putatively involved in glutamate synthesis but this enzyme was not detected in red mature fruits where glutamate accumulates (Sorrequieta et al., 2010). Considering the importance of glutamate in phloem sap, transfer of this amino acid from the source leaves to the maturing fruits could not be excluded. (Snowden et al., 2015) considered that GABA may be interconverted in Glu and Asp and provided evidences that these amino acids must be stored in the vacuoles. These authors identified SlCAT9 as a candidate protein for tonoplast transporter exporting GABA from the vacuole and importing Glu and Asp.

Aromatic amino acids also increase and are of special interest since they constitute precursor of flavor volatiles during the ripening process. Valine increased in relation to a stimulation of dihydroxy acid dehydratase (Mounet et al., 2009). MYB and bZIP transcription factors were shown to affect amino acid metabolism (Mounet et al., 2009). (Zhao et al., 2018) recently demonstrated that TAGL1, which play a major role in fruit development (see above), also directly influences fruit metabolism in relation to an increase in seven amino acids (tyrosine, glutamic acid, valine, phenylalanine, proline, leucine and isoleucine).

## Secondary Metabolism in Tomato Fruit

### Pigments and Flavonoids

The onset and progression of ripening in tomato is typically associated with changes in the external color of the pericarp, reflecting the accumulation of carotenoid and flavonoid pigments (Shinozaki et al., 2018b). Tomato fruits typically provide the principal dietary source of carotenoids in many Western diets (Carrari and Fernie, 2006). The characteristic red tomato color is a result of the accumulation of the carotenoid lycopene in both the fruit skin and pulp (Seymour et al., 2013; Borghesi et al., 2016; D’Ambrosio et al., 2018). During tomato ripening, the concentrations of carotenoids increase by between 10- and 14-fold, mainly due to the accumulation of lycopene (Fraser et al., 1994), which increases as the fruit matures (Tamasi et al., 2019). Alterations in the pigment accumulation patterns have also been observed in several spontaneously occurring tomato mutants (Carrari and Fernie, 2006); for example, the recessive mutant *high pigment* (*hp*) produces fruits with two times more carotenoids than wild-type fruits and increased levels of other antioxidants (Yen et al., 1997; Bino et al., 2005; Carrari and Fernie, 2006).

Carotenoid biosynthesis has been studied extensively in tomato, and major steps in the pathway have been identified (Seymour et al., 2013). Light signaling and plant hormones, particularly ethylene and auxins, have been identified as important regulators of carotenoid biosynthesis during tomato fruit ripening (Cruz et al., 2018). Almost all the enzymes acting in the carotenoid biosynthesis pathway have been cloned, and metabolic engineering approaches have been developed to enhance pigment quantity and quality (Carrari and Fernie, 2006; Alseekh et al., 2015; D’Ambrosio et al., 2018). The first committed step of carotenoid biosynthesis is the formation of phytoene, which is dependent on the catalytic activity of phytoene synthase. Phytoene then undergoes two desaturation reactions to form ζ-carotene, catalyzed by phytoene desaturase, which in turn is desaturated to neurosporene and finally lycopene. Lycopene is then either cyclized at both ends of the molecule by lycopene b-cyclase to form β-carotene, or cyclized at one end by lycopene b-cyclase and at the other by lycopene e-cyclase to form α-carotene. These cyclic carotenoids can then be converted to xanthophylls.

Tomatoes also accumulate semipolar metabolites, such as flavonoids, phenolic acids, and alkaloids, which are important health-promoting compounds (Bovy et al., 2007; Tohge and Fernie, 2015; Ballester et al., 2016; Tohge et al., 2017; Tamasi et al., 2019; Wang et al., 2019). To identify the genes responsible for their biosynthesis, QTL analyses were performed in different populations of introgression lines between *S. lycopersicum* and wild tomato species such as *S. chmielewskii* and *S. pennellii* (Alseekh et al., 2015; Ballester et al., 2016; Liu et al., 2016; Alseekh et al., 2017). The flavonoids represent a large family of low molecular weight polyphenolic secondary metabolites, which are grouped into several classes based on their aglycone structure (Bovy et al., 2007; Ballester et al., 2016). The main flavonoid classes are the flavones, flavonols, flavanones, flavanols, anthocyanidins, and isoflavones (Bovy et al., 2007; Tohge et al., 2017). More than 500 different forms of flavonoids are present in tomato, with the most major being the chalcone naringenin chalcone and various sugar conjugates of the flavonols quercetin and kaempferol, including rutin (Bovy et al., 2007; Ballester et al., 2016; Tamasi et al., 2019). In tomato fruits, the accumulation of flavonoids is restricted to the peel, with only traces found in the flesh, which comprises approximately 95% of the whole fruit (Schijlen et al., 2008; Bovy et al., 2010; Ballester et al., 2016). As a result, in a typical tomato cultivar such as Moneymaker, quercetin levels rarely go above 10 mg kg^‑1^ fresh weight (Bovy et al., 2010). Usually, cultivated tomatoes lack high levels of anthocyanins, while some wild tomato species (*S. chilense* and *S. cheesmaniae*) have much higher levels, giving a purple tone to the skin of certain organs (Seymour et al., 2013; Borghesi et al., 2016; Wang et al., 2019). The main phenylpropanoids found in tomato are chlorogenic and caffeic acids (Tamasi et al., 2019).

Flavonoids, along with other phenylpropanoids, are biosynthesized from phenylalanine. Three enzymes [phenylalanine ammonia lyase (PAL), cinnamate 4-hydroxylase (C4H), and 4-coumaroyl CoA ligase (4CL)] convert phenylalanine into 4-coumaroyl CoA, the activated intermediate for the various branches of phenylpropanoid metabolism (Zhang et al., 2015). Chalcone synthase (CHS) is the first enzyme involved in the phenylpropanoid/flavonoid pathway and converts 4-coumaroyl CoA into naringenin chalcone (Ballester et al., 2016; Tohge et al., 2017). Most of the biosynthetic genes involved in the flavonoid pathway and the transcription factors regulating them have been identified (Adato et al., 2009; Bovy et al., 2010; Ballester et al., 2016; Tohge et al., 2017; Li et al., 2018b; Wang et al., 2019). These insights have been used to develop genetic engineering strategies to increase the flavonoid contents of tomatoes, since this species accumulates limited amounts of phenolic antioxidants relative to its content of lipophilic antioxidants such as carotenoids (Carrari and Fernie, 2006; Bovy et al., 2007; Bovy et al., 2010; Zhang et al., 2015; Tohge et al., 2017; Wang et al., 2019).

Alkaloids are generally considered to be antinutritional factors in our diet (Friedman, 2002). Breeding efforts have focused on reducing their levels in foods, but some of these substances still remain in our daily diet (Friedman, 2002). More than 100 glycoalkaloids have been found to be present in the tomato clade in various tissues and accessions (Tohge and Fernie, 2015). The main alkaloids present in tomato are α-tomatine and dehydrotomatine, which are often concurrently analyzed as tomatine (Friedman, 2015; Ballester et al., 2016; Tamasi et al., 2019). Immature green tomatoes contain up to 500 mg of tomatine per kilogram of dry weight, while the levels in red tomatoes are much lower (up to about 5 mg kg^‑1^) (Friedman, 2015; Tamasi et al., 2019). The tomatine contents of cherry tomatoes (grape tomatoes, minitomatoes) are several fold greater than those of the larger standard tomato varieties (Friedman, 2015; Tamasi et al., 2019). In tomato fruits, the bitter-tasting α-tomatine is present at high levels in early developmental stages, but its levels decrease upon ripening due to its conversion into its acetyl glucosylated forms lycoperoside G and F or esculeoside A, which are not bitter (Tohge and Fernie, 2015; Ballester et al., 2016). Dehydrotomatine is 10 times less abundant than α-tomatine in immature fruits (Tamasi et al., 2019). Despite their negative impact on nutrition and their toxicity, glycoalkaloids found in Solanaceous plants, such as α-tomatine, and their hydrolysis products were shown to have anticancer properties (Friedman, 2015).

### Volatiles

Volatile metabolites biosynthesized during tomato ripening are responsible for fruit flavor and aroma (Carrari and Fernie, 2006; Ballester et al., 2016; Shinozaki et al., 2018b). More than 400 volatiles have been detected in tomatoes, but a smaller set of 15 to 20 are made in sufficient quantities to have an impact on human perception (Baldwin et al., 2000; Mathieu et al., 2009; Zanor et al., 2009). These volatile compounds are generally derived from various precursors, including fatty acids, carotenoids, and amino acids (Tieman et al., 2006; Zanor et al., 2009; Bauchet et al., 2017). The principal contributors to the ripe tomato flavor are cis-3-hexanal, cis-3-hexanol, hexanal, 3-methylbutanal, 6-methyl-5-hepten-2-one, 1-pentan-3-one, trans-2-hexanal, methyl salicylate, 2-isobutylthiazole, and β-ionone (Carrari and Fernie, 2006). There are differences of many orders of magnitude between the abundance of the various volatile compounds, with concentrations ranging from several micrograms per gram of fresh weight for the most abundant, such as (*Z*)-3-hexenal or hexanal, to nanograms per gram and even lower levels detected for β-damascenone or β-ionone (Rambla et al., 2014; Tieman et al., 2017). The levels of almost any volatile compound also vary substantially between varieties and accessions (Rambla et al., 2014). Modern commercial varieties contain significantly lower amounts of many of the important flavor chemicals than older varieties since it was not the focus of breeding programs (Tohge and Fernie, 2015; Bauchet et al., 2017; Tieman et al., 2017). Volatiles display a variable pattern of heritability, suggesting a high sensitivity to environmental conditions (Bauchet et al., 2017). Moreover, not all volatile compounds confer positive taste attributes to tomato (Carrari and Fernie, 2006). An example is the identification of *malodorous*, a wild tomato species allele affecting tomato aroma that was selected against during domestication (Tadmor et al., 2002).

QTL analyses, genome-wide association studies, and targeted metabolome quantifications were conducted in several cultivars and accessions of cultivated tomato, wild relatives, and inbred lines to identify tomato volatiles and their associated genetic loci (Saliba-Colombani et al., 2001; Tieman et al., 2006; Mathieu et al., 2009; Zanor et al., 2009; Alseekh et al., 2015; Ballester et al., 2016; Liu et al., 2016; Alseekh et al., 2017; Bauchet et al., 2017; Tieman et al., 2017). These studies revealed the complex and distinct regulation of metabolites in tomato subspecies (Rambla et al., 2014; Bauchet et al., 2017), demonstrating that there is ample genetic scope to improve the volatile composition of commercial varieties (Rambla et al., 2014).

As mentioned previously, several classes of volatiles exist in tomato. Volatiles derived from fatty acids constitute a class of compounds containing the most abundant volatiles produced in tomato fruits: the C6 volatiles 1-hexanol, (Z)-3-hexenal, (E)-2-hexenal, or hexanal, and the C5 volatile 1-penten-3-one (Rambla et al., 2014). These compounds are classified as green-leaf volatiles due to their characteristic fresh aroma of cut grass (Rambla et al., 2014). The production of these compounds increases as the fruit ripens (Klee, 2010). A second class are volatiles derived from amino acids. A significant number of volatile compounds considered important for the tomato aroma are derived from amino acids (Rambla et al., 2014). These volatiles can be grouped into two categories: phenolic and branched-chain compounds. Phenolic volatiles include a variety of compounds derived from the amino acid phenylalanine, while branched-chain volatiles have particularly low molecular weights and high volatility (Rambla et al., 2014). Additional classes are ester and terpenoid volatiles. Few esters are found in the volatile fraction of tomato (Rambla et al., 2014), while volatile terpenoids are among the most abundant volatiles in tomato vegetative tissues, but only a few of them, such as limonene, linalool, or α-terpineol, are present in the ripe fruit (Rambla et al., 2014), Volatile terpenoids can be classified into two groups, the monoterpenoids (C10) and sesquiterpenoids (C15), both of which are biosynthesized from the five-carbon precursors isopentenyl diphosphate and dimethylallyl diphosphate (Rambla et al., 2014). Carotenoid-derived volatiles are produced at low levels in ripe fruit but are important in our perception of tomato flavor due to their very low odor thresholds (Vogel et al., 2010; Rambla et al., 2014). This is particularly true for β-ionone or β-damascenone, which can be detected ortho-nasally at concentrations of 0.007 and 0.002 nL L^‑1^, respectively (Buttery et al., 1989). Volatile compounds first accumulate in a conjugated nonvolatile form, such as a glycoside, before being released during the ripening process (Rambla et al., 2014; Tohge and Fernie, 2015). The accumulation of the appropriate glycosidases in a separate subcellular location would allow the immediate liberation of high amounts of the aglycone when the enzyme and the conjugate glycosylated form come into contact with each other (Rambla et al., 2014).

## Effects of Abiotic Stress on Tomato Fruit Metabolism

Tomato is one of the most cultivated vegetable species but its productivity is impaired by a wide range of abiotic stresses (Gerszberg and Hnatuszko-Konka, 2017). The presence of adverse environmental factors like extreme temperatures, salinity or drought affects tomato yield as a consequence of reduced fruit number and fruit size but it also affects fruit quality (Moretti et al., 2010; Li et al., 2012; Gerszberg and Hnatuszko-Konka, 2017). It has been shown that moderate stress conditions may improve fruit quality through higher concentration of flavor compounds (Zheng et al., 2013; Albert et al., 2016a; Albert et al., 2016b). In several studies, the concentrations of sugars, organic acids, vitamin C, phenolic compounds and carotenoids increased in tomato fruits in response to water deficit, salinity, or heat (Saito et al., 2009; Patanè et al., 2011; Zushi and Matsuzoe, 2015; Albert et al., 2016b; Flores et al., 2016; Marsic et al., 2018). However, increased CO_2_ levels increased fruit production but decreased fruit quality (Mamatha et al., 2014). Nevertheless, metabolic modifications in tomato fruits in response to abiotic stress may be cultivar-dependent (Sánchez-Rodríguez et al., 2012a; Sánchez‐Rodríguez et al., 2012b; Zushi and Matsuzoe, 2015; Albert et al., 2016b; Albert et al., 2016a; Flores et al., 2016; Marsic et al., 2018). **Table 1** summarizes some recent studies regarding the impact of abiotic stress occurring during plant growth on primary and secondary metabolism of tomato fruits. Modification of fruit metabolism was mainly investigated in response to salinity and drought (**Table 1**). The effect of salinity was investigated in various cultivars under hydroponic culture with NaCl concentrations varying between 0 to 100 mM and salinity overall increased the concentrations of sugars, organic acids, amino acids, pigments and antioxidants (Saito et al., 2009; Schnitzler and Krauss, 2010; Zushi and Matsuzoe, 2015; Flores et al., 2016; Marsic et al., 2018). The effect of drought was investigated under both greenhouse and field conditions. Most studies reported an increase in sugars and organic acids in response to drought in a wide range of tomato accessions (Patanè et al., 2011; Murshed et al., 2013; Zheng et al., 2013; Shao et al., 2014; Albert et al., 2016b; Albert et al., 2016a) while others reported less strong effects (Atkinson et al., 2011; Sánchez‐Rodríguez et al., 2012; Wei et al., 2018). The effect of drought on the concentration of secondary metabolites was more cultivar-dependent (Atkinson et al., 2011; Sánchez‐Rodríguez et al., 2012). In contrast to salinity and drought, heat mainly decreased the concentration of pigments and ascorbic acid in tomatoes (Li et al., 2012; Hernández et al., 2015) and increased CO_2_ levels decreased carotenoid, polyphenol and flavonoid concentrations but increased ascorbic acid concentration in tomatoes (Mamatha et al., 2014). All these compounds play an important role in the final nutritional and commercial quality of tomato and depend on genetic, environmental, agronomic and post-harvest factors (Flores et al., 2016). Several studies based on the influence of these factors on fruit composition have been carried out with the aim of increasing tomato quality (Flores et al., 2016).

**Table 1:** Effect of abiotic stress occurring during plant growth on primary and secondary metabolite production in tomato fruits.

Metabolites	Salinity	Drought	Heat	Cold	CO_2_ increase
**Primary metabolites**					
**Sugars**					
Soluble solid Content	↑	↑		=	↓(↑)
Total soluble Sugars	↑	↑(=*)	↑	=	↑
Fructose	↑	↑(↓, =*)			
Glucose	↑	↑(↓, =*)			
Saccharose	↑	(=*)			
**Organic acids**					
Citric acid	↑(=*)	↑(=*)		=	↓
Malic acid	↑(=*)	↑(=*)		=	↓
Glutamic acid	↑(=*)				
Quinic acid	↑(=*)				
**Amino acids**					
Arginine	↑*				
Histidine	↑*				
Isoleucine	↑*				
Threonine	↑				
Serine	↑				
Proline	↑				
Phenylalanine	↑				
**Secondary metabolites**					
**Pigments**					
Carotenoids	↑(=)	↑(=*)	↓		↓
Lycopene	↑*(=)	↑(↓*)	↓		↓
β-carotenoid	↑	↑(↓*)	↓=		
**Antioxydants**					
Total		↑			
Polyphenols	↑*	↑(=*)			↓
Flavonoids	↑*	↑(=,↓*)			↓
Ascorbic acid	↑(=*)	↑(=*)	↓		↑=
References	(Saito et al., 2009; Schnitzler and Krauss, 2010; Zushi and Matsuzoe, 2015; Flores et al., 2016; Marsic et al., 2018)	(Atkinson et al., 2011; Patanè et al., 2011; Sánchez‐Rodríguez et al., 2012; Murshed et al., 2013; Zheng et al., 2013; Shao et al., 2014; Albert et al., 2016a; Albert et al., 2016b; Wei et al., 2018)	(Li et al., 2012; Hernández et al., 2015)	(Kläring et al., 2015)	(Moretti et al., 2010; Mamatha et al., 2014; Wei et al., 2018)

↑ : Increase; ↓: Decrease; =: No modification; *Cultivar-dependent; () Effect observed only in one study or few cultivars.

In addition to the environmental conditions to which plants are subjected during their growth, post-harvest conditions may also affect fruit quality and metabolism. The impact of low temperature storage on tomato quality has been extensively investigated (Sevillano et al., 2009; Luengwilai et al., 2012; Cruz-Mendívil et al., 2015; Wang et al., 2015; Raffo et al., 2018; Zhang et al., 2019). Among others, early harvesting and cold storage negatively affect tomato flavor and decrease the levels of aroma compounds (Wang et al., 2015; Raffo et al., 2018). Indeed, metabolomics data showed that 7 amino acids, 27 organic acids, 16 of sugars and 22 other compounds had a significantly different content in cold-stored tomatoes and transcriptomics data showed 1735 differentially expressed genes due to cold storage (Zhang et al., 2019). Some pre-treatments have been proposed to improve tomato fruit resistance to cold stress such as ozone exposition, high CO_2_ treatment, UV-C hormesis, oxalic acid application and heat treatment (Moretti et al., 2010; Luengwilai et al., 2012; Mattos et al., 2014; Charles et al., 2015; Cruz-Mendívil et al., 2015; Li et al., 2016b; Sangwanangkul et al., 2017; Raffo et al., 2018). These treatments provide protection from chilling in part by altering levels of fruit metabolites (Luengwilai et al., 2012; Wang et al., 2015; Sangwanangkul et al., 2017).

## Conclusions

In this review, we focused on the tomato fruit development and metabolism. Tomato has long been the model for the study of fleshy fruits and the emergence of “omics” approaches (phenomics, genomics, transcriptomics, proteomics, and metabolomics) has largely contributed to improve our understanding of the genetic, hormonal and metabolic networks that govern tomato fruit development and metabolism. Tomatoes are climacteric fruits with high level of health-promoting compounds. As important as yield improvement and stress resistance, enhancement of tomato fruit quality has gained extensive attention. Improvement of tomato flavor and quality is a challenge for the coming years. The sequencing of tomato genome and genome-wide association studies provide genetic insights into the genetic control of tomato flavor and gives a roadmap for flavor improvement. Moreover, several techniques can now be exploited for breeding superior tomato varieties in the context of current changing climatic conditions.

## Author Contributions

MQ and SL designed the outline of the manuscript. MQ, SL, FY-L, TA, and J-PM contributed to writing and revisions of the manuscript. SB and RB-G contributed to figure design and revisions of the manuscript. All authors read and approved the final manuscript.

## Funding

This work was supported by funding from the Belgium “Fonds National de la Recherche Scientifique (FRS-FNRS)” (grant no. CDR J.0136.19).

## Conflict of Interest

The authors declare that the research was conducted in the absence of any commercial or financial relationships that could be construed as a potential conflict of interest.
